# Benzo[*a*]pyrene—Environmental Occurrence, Human Exposure, and Mechanisms of Toxicity

**DOI:** 10.3390/ijms23116348

**Published:** 2022-06-06

**Authors:** Bożena Bukowska, Katarzyna Mokra, Jaromir Michałowicz

**Affiliations:** Department of Biophysics of Environmental Pollution, Faculty of Biology and Environmental Protection, University of Lodz, Pomorska Str. 141/143, 90-236 Lodz, Poland; katarzyna.mokra@biol.uni.lodz.pl (K.M.); jaromir.michalowicz@biol.uni.lodz.pl (J.M.)

**Keywords:** benzo[*a*]pyrene, polycyclic aromatic hydrocarbons, metabolism, genotoxicity, carcinogenicity

## Abstract

Benzo[*a*]pyrene (B[*a*]P) is the main representative of polycyclic aromatic hydrocarbons (PAHs), and has been repeatedly found in the air, surface water, soil, and sediments. It is present in cigarette smoke as well as in food products, especially when smoked and grilled. Human exposure to B[*a*]P is therefore common. Research shows growing evidence concerning toxic effects induced by this substance. This xenobiotic is metabolized by cytochrome P450 (CYP P450) to carcinogenic metabolite: 7β,8α-dihydroxy-9α,10α-epoxy-7,8,9,10-tetrahydrobenzo[*a*]pyrene (BPDE), which creates DNA adducts, causing mutations and malignant transformations. Moreover, B[*a*]P is epigenotoxic, neurotoxic, and teratogenic, and exhibits pro-oxidative potential and causes impairment of animals’ fertility. CYP P450 is strongly involved in B[*a*]P metabolism, and it is simultaneously expressed as a result of the association of B[*a*]P with aromatic hydrocarbon receptor (AhR), playing an essential role in the cancerogenic potential of various xenobiotics. In turn, polymorphism of *CYP P450* genes determines the sensitivity of the organism to B[*a*]P. It was also observed that B[*a*]P facilitates the multiplication of viruses, which may be an additional problem with the widespread COVID-19 pandemic. Based on publications mainly from 2017 to 2022, this paper presents the occurrence of B[*a*]P in various environmental compartments and human surroundings, shows the exposure of humans to this substance, and describes the mechanisms of its toxicity.

## 1. General Information

**Figure 1:**
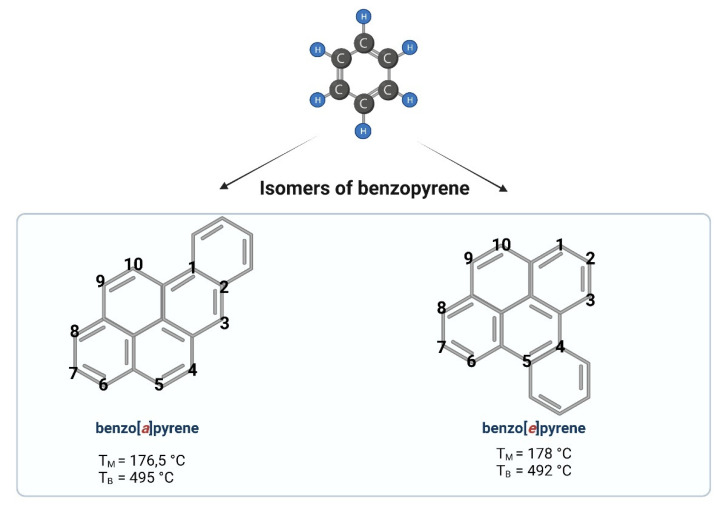
Chemical structures of benzopyrene. Created with BioRender.com (accessed on 12 April 2022).

Benzo[*a*]pyrene (B[*a*]P) is one of the representatives of aromatic hydrocarbons. Having five benzene rings, B[*a*]P belongs to a group of polycyclic aromatic hydrocarbons (PAHs). Depending on the place of the fifth benzene ring attachment, we can distinguish B[*a*]P (a single benzene molecule attached at the bond *a*) or benzo[*e*]pyrene (a single benzene molecule attached at the bond *e*) (Figure 1). A single molecule of benzene is liquid, while benzopyrene is solid. PAHs containing two or more fused benzene rings form stable molecule structures of a high hydrophobic nature [1].

B[*a*]P and other PAHs have been shown to be formed during the burning of fossil fuels, wood, and other organic materials. B[*a*]P has been detected in high levels in cigarette smoke, diesel exhaust, charcoal-based foods, as well as industrial wastes [2]. It is therefore a substance that is formed as a by-product in various thermal processes. 

The exposure to B[*a*]P in recent times is more common than ever before. Sources of B[*a*]P emissions are mostly anthropogenic, and to a lower extent natural, including wildfires and volcanic eruptions [3].

In mammals, B[*a*]P is readily absorbed after inhalation, oral administration, and through the skin [4]. The main sources of human B[*a*]P exposure are contaminated food and the air [5].

An important source of exposure to B[*a*]P is tobacco smoke. B[*a*]P concentrations in the side stream of cigarette smoke was shown to be in the range of 52 to 95 ng/cigarette—over three times higher than in mainstream smoke [6]. High concentrations of B[*a*]P, and particularly its metabolite—7β,8α-dihydroxy-9α,10α-epoxy-7,8,9,10-tetrahydrobenzo[*a*]pyrene (BPDE)—have been determined in smokers. Susanto et al. [7] detected a higher level of BPDE protein adducts in smokers (8.87–33.55 ng/mL) compared to nonsmokers (3.87–13.27 ng/mL). BPDE DNA adducts were also determined in the placenta of smoking mothers [8]. In smokers (compared to nonsmokers), the exposure to B[*a*]P present in tobacco smoke leads to a prolonged gestation period, earlier mean menopausal age, altered ovarian steroidogenesis, and ovarian reserve depletion [9].

## 2. Occupational Exposure to PAHs, Including B[*a*]P

B[*a*]P is usually present in the highest concentrations in the mixture of PAHs (emitted during combustion and other technological processes) to which workers in many branches of industry are exposed. Therefore, when evaluating the correlation between the incidence of a given disease (including people representing specific occupational groups) and the exposure to PAHs mixtures, it is justified to relate this association indirectly to B[*a*]P.

Occupational exposure to PAHs, including B[*a*]P, occurs mainly through inhalation and dermal contact. The highest mean level of PAHs has been determined in aluminum production (Söderberg process) with concentrations up to 100 μg/m^3^. Average levels of PAHs are detected in roof coverings and pavements (e.g., 10–20 μg/m^3^), and the lowest (i.e., 1 μg/m^3^ or below) have been noted in coal liquefaction, coal-tar distillation, wood impregnation, chimneys, and power plants [6].

Saleh et al. [10] assessed differences in the PAHs, including B[*a*]P concentrations, in samples of air inhaled by occupationally exposed and nonexposed groups inhabited in Makkah (Saudi Arabia). They detected B[*a*]P in the mean concentration of 0.082 ± 0.032 ng/m^3^ in occupationally exposed workers (bus and truck drivers, police officers, etc.), while its mean concentration in unexposed group was 0.044 ± 0.006 (Table 1). Interestingly, they observed a positive correlation between increased B[*a*]P exposure and serum p51 and p21 proteins levels that are considered to be implicated in tumor progression, invasion, and metastasis.

In another work, Petit et al. [11] have recently evaluated the incidence of lung cancer risk related with PAHs exposure in various industries using atmospheric B[*a*]P level as a surrogate value. They analyzed a total of 93 exposure groups, which belonged to nine branches of industry. They have shown that 30% of exposure groups were above the maximum risk level of the European Union (10^−4^).

## 3. Sources of Human Exposure to B[*a*]P

Accumulation of B[*a*]P and other PAHs on dust particles, as well as in fats, is associated with their high hydrophobicity. B[*a*]P accumulates in plants and the adipose tissue of animals and is strongly attached to the organic fraction of soil but does not penetrate deeper layers of soil. Therefore, it is poorly taken up by the plant root system [19].

Recently, Sushkova et al. [20] reported that B[*a*]P concentrations in plants depended on the initial B[*a*]P level in the soil. It was observed that growing spring barley in the B[*a*]P-spiked soils accumulated this compound.

The estimated half-lives for B[*a*]P are less than 1–6 days in the atmosphere, less than 1–8 h in water, but more than 5–10 years in sediments and above 14–16 months in soil (for complete degradation) [21].

### 3.1. Air

#### 3.1.1. Outdoor Air

PAHs, including B[*a*]P, are significant components of air pollution. Most emissions of PAHs derive from human sources, i.e., fossil fuels pyrolysis, incomplete combustion, and burning of biomass [22]. PAHs are substantial parts of semivolatile organic substances. PAHs of low molecular weight (2–3 rings) are most commonly detected in the gas phase due to their low boiling points, whereas PAHs of high molecular weight, including B[*a*]P (4–6 rings), are mainly adsorbed on the particulate phase and exhibit stronger carcinogenicity [23].

B[*a*]P is considered to be an indicator for the determination of a PAHs group; therefore, it is an important tool employed in the monitoring of the environment, including the evaluation of air quality [24]. The European Union (EU) decided that B[*a*]P level in the air must not exceed 1 ng/m^3^, while higher levels of this substance have been reported in various areas of the world, including countries of the European Union [25,26]. The mean levels of various PAHs in the outdoor air of urban areas are usually from about one up to several dozen ng/m^3^. The highest B[*a*]P levels, reaching several dozens of nanograms per cubic meter, were found in road tunnels and in big cities, which intensively utilize coal and other fuels to heat houses [24,26]. It has been estimated that 20% of the European population is exposed to B[*a*]P at the concentrations that exceeded the EU annual admissible value of 1 ng/m^3^, while 7% of people live in regions in which B[*a*]P amounts are under the tolerable risk level of 0.12 ng/m^3^ [12].

For instance, in France (taken from studies from 1990 to 2011), the concentration range of B[*a*]P in the air was from below 1 ng to 2.49 ng/m^3^ [13], while over several regions of the Iberian Peninsula its level exceeded the European target value of 1 ng/m^3^ [22]. Similarly, in Genoa (Italy), in areas along heavily trafficked streets, the average B[*a*]P concentration was determined to be 2 ng/m^3^, while on a building’s roof, 300 m from a coke oven, its mean level was detected to be much higher, at 14 ng/m^3^ [16].

Among European countries, Poland is one with poor air quality, where high B[*a*]P levels are detected in the outdoor air, particularly during the heating season. Flaga-Maryańczyk and Baran-Gurgul, (2021) reported changes in atmospheric B[*a*]P levels in Cracow and nearby localities (Tarnow, Nowy Sacz) in the years 2011–2020 [17]. The scientists determined levels that repeatedly exceeded standards for B[*a*]P, which were in the range from 4 to 10 ng/m^3^, 4 to 6 ng/m^3^, and 10 to 11 ng/m^3^ in Cracow, Tarnów, and Nowy Sącz, respectively. Similarly, in the air of Linzhou (China), B[*a*]P was detected in a very high concentration range, from 5.1 to 20.2 ng/m^3^ [18].

Mahasakpan et al. [14] assessed Thailand’s air quality, revealing that mean B[*a*]P concentrations (adsorbed at particle phase) during partial and strong haze periods were 0.052 and 0.095 ng/m^3^ in PM 2.5 fraction, respectively, which was 6 and 11 times higher in comparison to the normal period (0.009 ng/m^3^). Disturbingly, B[*a*]P adsorbed on PM1 (the particles that can penetrate deeply into the respiratory tract and enter the circulatory system) contributed to 74.0–91.1% of B[*a*]P-PM 2.5 fraction.

#### 3.1.2. Indoor Air

PAHs’ emission, as a result of the combustion of numerous materials in indoor environments, is a great danger for humans, particularly in less developed countries. Indoor air pollution has been shown to be strongly linked with infections of the respiratory tract, as well as nasopharyngeal and lung cancer [27]. It has been estimated that the pollution of indoor air is responsible for 3.5–4 million deaths each year worldwide, with 1 million deaths in China [28].

The study of Feng et al. [29] aimed to assess various PAHs levels in gaseous samples collected from indoor kitchens, during cooking, in 35 rural houses in North China. They determined a high concentration of B[*a*]P both in the gaseous and particulate phases, which were 14.3 ± 23.0 ng/m^3^ and 6.7 ± 17.4 ng/m^3^, respectively (Table 2). The authors suggested that that kind of combusted fuel and ventilation effectiveness had a significant effect on B[*a*]P levels. In another study, Li et al. [30] assessed the presence of PAHs emitted from different cooking activities in Yucheng City (China). They observed that total mean PAHs levels were from 105 ± 65 ng/m^3^ to 783 ± 499 ng/m^3^, and were the highest in the indoor air where cafeteria frying occurred. They also observed that cooking methods and fat contents in raw materials mainly determined PAHs emissions. As the study showed, the B[*a*]P level was much higher for the oil-based (25.8 ± 10.6 ng/m^3^, meat roasting) than the water-based (7.3 ± 4.6 ng/m^3^, cafeteria boiling) cooking activities. Interestingly, Cui et al. [31] analyzed indoor air pollution in the Jokhang Temple (Tibet) and detected B[*a*]P at a mean concentration of 18.5 ± 4.3 ng/m^3^, which exceeded the maximum permissible risk value of 1 ng/m^3^ by nearly twenty times. The authors of the study suggested that high PAHs, including B[*a*]P levels, was due to the intense burning of plant material, the dense flow of people, and poor ventilation in the building.

Children are the most sensitive to indoor air pollutants because of their not-fully developed respiratory and immune systems. Liu et al. [32] observed that in classrooms in schools in Shanxi Provence (China), the concentrations of B[*a*]P increased from 0.05 ng/m^3^ (in the nonheating season) to as high as 10.3 ng/m^3^ (in the heating season), which showed that children were highly exposed to this compound in winter. The analysis also revealed that the combustion of coal and gasoline mainly contributed to B[*a*]P presence in the indoor environment of tested buildings. In another study, Alghamdi et al. [33] determined classroom, dust-bound PAHs, including B[*a*]P, in Jeddah’s schools in Saudi Arabia. The authors detected B[*a*]P at a very high mean concentration of 163.87 ± 68.53 ng/m^3^, noting that mean PAHs levels were higher in classrooms in urban schools as compared to suburban and residential schools. Moreover, based on incremental lifetime cancer risk and total lifetime cancer risk calculations, they suggested that dibenz[*a,h*]anthracene and B[*a*]P mainly contributed to cancer development in children in all schools tested. Similarly, the study conducted in Poland (Silesia) showed exceeded B[*a*]P levels in indoor samples collected in naturally ventilated kindergartens. The mean B[*a*]P level was estimated to be 3.7 ± 0.8 ng/m^3^, and its content was affected by the usage of gas and coal stoves [34].

Huge concentrations of PAHs, including B[*a*]P, are often detected in the indoor air of workplaces. Sen et al. [35] assessed the exposure of iron foundry workers (Shimoga, India) to PAHs, including B[*a*]P. They detected mean concentrations of total PAHs, which were from 23.48 μg/m^3^ at the melting section to 82.64 μg/m^3^ at the molding section, while mean and maximum B[*a*]P levels were determined to be as high as 7.20 ± 1.11 μg/m^3^ and 45.37 µg/m^3^, respectively. High mean concentrations of B[*a*]P, at levels of 14 μg/m^3^ and 3.3 μg/m^3^, were also determined in the indoor air of aluminum manufacturing factories and in coke oven facilities, respectively [36,37].

### 3.2. Surface Water

B[*a*]P has also been detected in surface water. Direct deposits of B[*a*]P in surface water are important inputs to the environment. PAHs, including B[*a*]P, which are deposited on the urban ground, are then washed out into waterways via erosion and surface run-off. The low solubility of B[*a*]P in water ecosystems results in its binding with organic matter, which leads to its occurrence in the hydrosphere [38].

A study performed in India showed that B[*a*]P content in river water reached 8.61 ng/L, and the total level of 17 PAHs was 157.96 ± 18.99 ng/L [39]. He et al. [38] also studied water in 44 Chinese lakes and determined levels of B[*a*]P from 0.07 to 2.26 ng/L.

Chen et al. [40] assessed PAHs, including B[*a*]P, levels in the surface water of the Danshui River basin by collecting samples from the Karst River in Hubei (central China). They determined mean total PAHs and B[*a*]P levels for 26.2 ng/L and 1.37 ng/L, respectively. In another study, Hayakawa et al. [41] analyzed PAHs, including B[*a*]P, in surface waters in the Southeast Sea (Japan). They detected 13 PAHs (3–6 rings) in the dissolved phase (DP) and 12 PAHs in the solid phase (PP), with total PAHs levels (DP + PP) ranging from 6.83 to 13.81 ng/L. Moreover, B[*a*]P in DP and PP phases was determined to be in the average levels of 0.12 ± 0.078 ng/L and 0.076 ± 0.037 ng/L, respectively. The authors concluded that a pyrogenic origin and a mixed type of pyrogenic and petrogenic origins were major contributors to the DP-PAHs and PP-PAHs, respectively.

### 3.3. Soil

Li et al. [42] showed that the spatial distribution of PAHs and B[*a*]P produced by industry impact the surrounding quality of soil. They pointed to following sources: combustion of coal (40.77%), exhausts from vehicles (32.94%), combustion of biomass (14.89%), and cooking (11.40%). Coal combustion and cooking were the prevalent sources (52.17%) of PAHs, as well as carcinogenic risk (46.48%) assessed by B[*a*]P toxic equivalent level in soil. The authors concluded that coal combustion mainly contributed to PAHs contamination and health risk in Taiyuan soil.

B[*a*]P levels in soil was assessed in Poland from 1993 to 1994. B[*a*]P concentrations in soil ranged from <0.0001 to 3.030 mg/kg, with the highest associated with motor vehicles and hand-stocked residential furnaces [43]. In 2011 and 2012, Wydro et al. [44] determined slightly lower B[*a*]P levels in soils collected in Bialystok, Poland. The concentration of B[*a*]P was from 0.3 to 0.9 mg/kg of soil. B[*a*]P was also found in the concentration range from 0.28 to 5.50 mg/kg in municipal soil in Cleveland [45]. In another study, B[*a*]P was determined to be in a mean value of 2.31 ± 1.13 μg/kg in soil from greenhouse crops in the Antalya Aksu region in Turkey [46] (Table 3).

Lee et al. [56] assessed PAHs’ distribution in the sediments and fish collected from 30 major rivers in Taiwan. They showed that the total concentrations of 26 PAHs were from 0.016 to 7.44 mg/kg dw and from 0.096 to 5.51 mg/kg ww in sediments and in fish, respectively, while the levels of B[*a*]P in sediments were from 0.01 to 1.68 μg/kg dw. It was observed that the composition of PAHs in sediments was different from that found in fish, showing that sediments are not a prevalent source of PAHs in fish. The bioaccumulation of different PAHs in fish depended on fish species, as well as on the environmental contamination level, content of lipids, living pattern, and trophic level of each fish species.

#### Biodegradation B[*a*]P by Microorganisms

B[*a*]P degradation in the environment by various micro-organisms has been widely studied and became very popular in respect to the advantage of a high level of microbial diversity and neutrality toward the environment. The enzymes produced by micro-organisms, such as cytochrome P450, oxygenases, and hydrolases, have been shown to degrade B[*a*]P [57].

Strains resistant to B[*a*]P that contribute significantly to its biodegradation have been isolated. Recently, Nzila et al. [58] described the strain 10SBZ1A as a *Staphylococcus haemoliticus* species that degrades B[*a*]P. Bioremediation of B[*a*]P by micro-organisms is associated with the degradation of this toxicant to less harmful products, including CO_2_ (mineralization). Only a few microbes, such as *Mycobacterium* sp., *Mycobacterium vanbaalenii*, *Stenotrophomonas maltophilia*, *Sphingomonas yanoikuyae JA*, *Mesoflavibacter zeaxanthinifaciens*, *Bacillus licheniformis*, *Bacillus subtilis*, *Novosphingobium pentaromativorans,* and *Ochrobactrum* sp. have been shown to degrade B[*a*]P in nonhalophilic conditions.

B[*a*]P degradation by micro-organisms is associated with various factors, i.e., B[*a*]P concentration and its bioavailability, as well as properties of soil. Recently, some technologies, e.g., bioaugmentation and biostimulation, have been developed to accelerate the rate of degradation of B[*a*]P. Furthermore, advanced technologies, such as omics and nanotechnology have provided new opportunities for more efficient microbial degradation of B[*a*]P and other PAHs [57].

On the other hand, biodegradation of PAHs in the environment also adversely affects bacterial organisms. During biodegradation, more toxic metabolites may be formed, which can damage the biological membranes of micro-organisms, penetrate the organisms, and impair their vital functions [59].

### 3.4. Food Contamination

Consumption of food contaminated with B[*a*]P, which is created as a result of food preparation and processing during its baking, frying, grilling, or smoking (high temperature processes), is an important source of this substance for humans. B[*a*]P commonly occurs in many food products [60], such as olive oil (2.19 ± 0.2 ppb), Alwana olive oil (31.3 ± 0.3 ppb) [61], milk (0.06–2.09 μg/kg), and meat/fish-based baby foods (0.00–1.66 μg/kg) [62]. B[*a*]P has also been determined in traditionally smoked goat cheeses, such as Wallahian-style cheese (0.85 ± 0.255 μg/kg) or smoked “Ritta” (17.0 ± 5.10 μg/kg) [63], charcoal-grilled chicken with and without marinating at 270 °C (1.19 ± 0.31 μg/kg; 2.22 ± 0.13 μg/kg) [64], oysters (1.26 ± 1.22 μg/kg), mussels (0.24 ± 0.18 μg/kg), fresh shellfish (0.31 ± 0.42 μg/kg) [65], Iranian bread samples (mean 0.1 μg/kg) [66], and cucumbers (4.35 ng/kg) [30], but not in chocolate [67].

Kazerouni et al. [68] estimated the daily intake of B[*a*]P in a variety of foods in 228 subjects in the metropolitan area of Washington, D.C. The highest B[*a*]P concentrations were found in grilled/barbecued very well-done steaks (4.15 μg/kg), hamburgers (0.9 μg/kg), and grilled/barbecued (0.9 μg/kg) well-done chicken with skin. B[*a*]P concentrations in nonmeat products were generally low. However, some grains and vegetables (e.g., kale, vegetable cabbage) showed B[*a*]P concentrations up to 0.5 µg/kg. It was found that in the studied population, grilled/barbecued meat and bread/cereal/grain constituted 21 and 29 percent of mean daily consumption of B[*a*]P, respectively.

#### Drinking Water

According to European Union (EU) standards, the admissible total concentration PAHs level in drinking water is set to be 200 ng/L [69], while the concentration limit for the total of four PAHs (B[*b*]F, B[*k*]F, B[*ghi*]P, and I[*cd*]P) is 100 ng/L, and for B[*a*]P it is 10 ng/L [70].

The concentrations of B[*a*]P in drinking water in Poland in Łódź city was from nondetected (ND) to 16 ng/L, and in Tomaszów Mazowiecki, it was from ND to 18 ng/L. In other cities, such as Bydgoszcz, Gdansk, Katowice, Cracow, Warszawa, and Lublin, mean concentrations of B[*a*]P were in the range from 4.00 to 8.33 ng/L. In all tested drinking water samples, the sum of eight PAHs’ concentration was less than 200 ng/L, as recommended by the World Health Organization (WHO) [71].

Aygun and Bagcevan [72] assessed B[*a*]P levels in samples of tap water collected from Sinop, Ordu, Çorum, Amasya, Giresun, and Kastamonu (Turkey). The mean B[*a*]P level for the abovementioned locations were from 0.11 ± 0.08 to 0.97 ± 0.75 ng/L. The sum of the concentrations of PAHs and B[*a*]P in drinking water were determined to be below the limits of 100 ng/L and 10 ng/L, respectively, as it was defined in the Regulation on Water Intended for Human Consumption. The detected concentrations were also below the limits established by the WHO and Turkish legislation.

## 4. Metabolism of B[*a*]P

B[*a*]P metabolism in humans was studied by [73]. The researchers gave B[*a*]P a microdose orally to humans and observed its rapid uptake (T_max_, 0.5–1 h), complex metabolism (predominantly at the B[*a*]P bay region), and fast removal (mean half-life—46.5 ± 58.2 h) from the body. Parent [14C]-B[*a*]P was a minor component in plasma as compared to the sum of metabolites, and decreased along with the dose. Pharmacokinetic parameters for almost all metabolites showed a linear dose–response correlation. They also observed interindividual differences in pharmacokinetics within doses of 25–250 ng.

**Figure 2:**
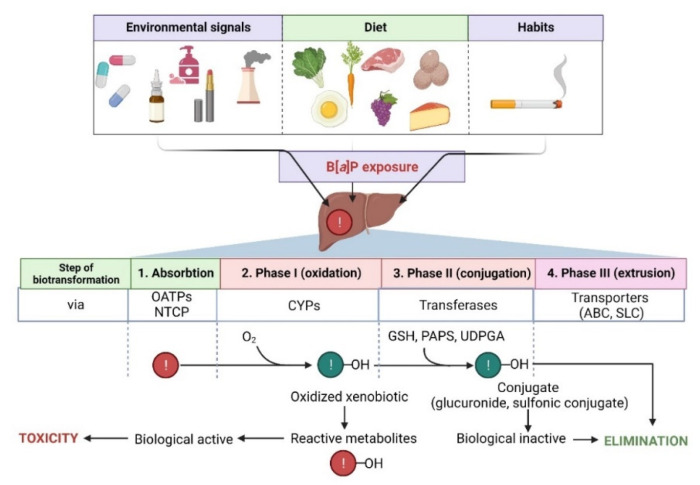
Steps in the biotransformation of B[*a*]P. Detoxification of B[*a*]P predominantly occurs with the participation of several cytochrome P450 (CYPs) isoforms. During phase I, xenobiotic is oxidized to reactive metabolites that may exhibit biological activity. In phase II, metabolites are conjugated with transferases (GSH, reduced glutathione; PAPS, 3′-phosphoadenosine 5′-phosphosulfate; UDPGA, uridine diphosphate-glucuronic acid) and converted to more hydrophilic and biologically inactive forms. Then, metabolites are eliminated from the cell via specialized transporters (ABC, ATP-binding cassette transporter; SLC, solute carrier). NTCP, sodium/taurocholate cotransporting polypeptide; OATPs, organic anion transporting polypeptides—biological systems enabling the transport of xenobiotics to the cell [74,75]. Created with BioRender.com (accessed on 12 April 2022).

The metabolism of B[*a*]P involves several phases, as it also is in the case of many other hydrophobic xenobiotics (Figure 2).

The first phase of biotransformation of PAHs, including B[*a*]P, involves the activity of cytochrome P450 (CYP1 family) and of microsomal epoxide hydrolase. During the first phase, PAHs are transformed to some phenols (hydroxy derivatives), phenol diols, dihydrodiols, quinones, and reactive-diol-epoxides enantiomers. During PAHs transformation, ROS are also produced as by-products of these reactions.

**Figure 3:**
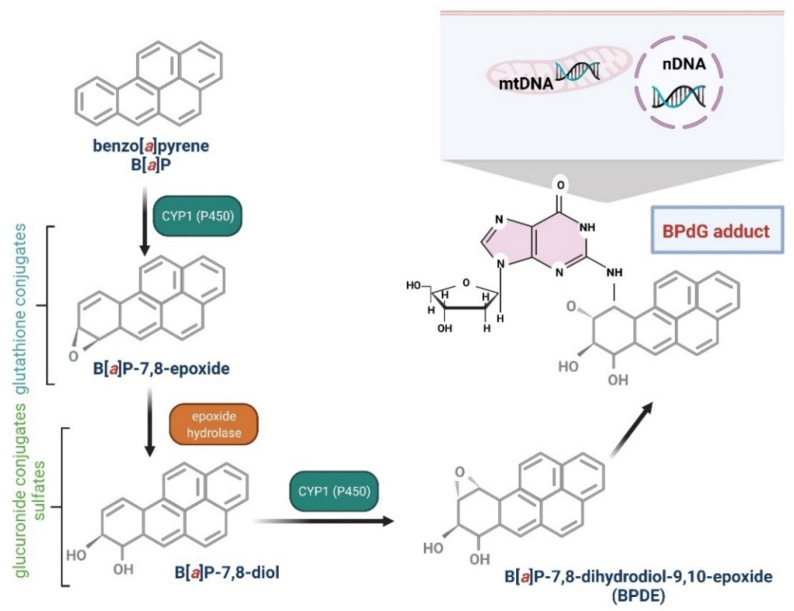
Biotransformation of B[*a*]P into mutagen. B[*a*]P in the I phase of detoxification goes through several stages, giving various derivatives, including B[*a*]P-7,8-diol-9,10-epoxide, that are able to react with DNA guanine. The reaction results in the formation of adducts with DNA (mtDNA, mitochondrial DNA; nDNA, nuclear DNA) and mutations [80,81]. Created with BioRender.com (accessed on 12 April 2022).

Several enzymes acting within cytochrome P450, including CYP1A1, CYP1A2, and CYP1B1, have been shown to be implicated in B[*a*]P oxidation, while CYP1A1 is considered to be the most active in mammals [76,77]. B[*a*]P epoxidation by P450 at the 7,8 positions has been found to be one of the most dangerous reactions, which leads to the formation of B[*a*]P toxic metabolites. Interestingly, B[*a*]P oxidation at the 4 and 5 positions leads to the creation of an inactive metabolite, which is eliminated from the organism [78]. As mentioned above, CYP1A1 can convert B[*a*]P to B[*a*]P-7,8-epoxide, which (in the presence of epoxide hydrolase) is transformed to (+/−)-B[*a*]P-trans-7,8-dihydrodiol (DHD). B[*a*]P-7,8-DHD is a substrate for the reaction of the second CYP-dependent oxidation, which forms the final carcinogenic metabolite—7β,8α-dihydroxy-9α,10α-epoxy-7,8,9,10-tetrahydrobenzo[*a*]pyrene (BPDE). It has been shown that in the cell nucleus, diol-epoxides can bind covalently to DNA, creating deoxyguanosine-DNA adducts that may lead to erroneous replication and mutagenesis [76,78] (Figure 2). Metabolism of B[*a*]P may therefore lead to the production of electrophilic metabolites exhibiting a carcinogenic potential. Cytochrome P4501A1 (CYP1A1) is believed to play the most important role in the pro-carcinogenic B[*a*]P activation necessary for the formation of DNA adducts. Shiizaki et al. [79], in a review paper, described factors that could affect the formation of B[*a*]P-DNA adducts and hypothesized that CYP1A1 was a key enzyme in the production of (BPDE), the major carcinogenic intermediate of B[*a*]P (Figure 3).

AhR activators, such as 2,3,7,8-tetrachloro-dibenzo(p)dioxin, may potentially increase B[*a*]P toxicity through induction of the *CYP1A1* gene. At the same time, it is believed that inhibitors of CYP1A1, including its substrates, may reduce the toxicity of B[*a*]P [76,79].

Phase I metabolism of B[*a*]P may not only be mediated by CYP but also by flavin-containing mono-oxygenases, NAD(P)H: quinone oxidoreductases, amine oxidases, esterases, alcohol dehydrogenases, and peroxidases [76]. Prostaglandin H synthase and lipoxygenase also participate in metabolic transformations of B[*a*]P, being responsible for the formation of highly reactive, oxygen-free radicals [82]. In the presence of polyunsaturated fatty acids, including linolenic and arachidonic acid, B[*a*]P undergoes oxidative transformations, and the resulting oxidized B[*a*]P and lipid peroxidation products demonstrate mutagenic potential. Thus, products of B[*a*]P oxidation and polyunsaturated fatty acid peroxidation may participate in mutagenesis and may also induce carcinogenesis [83].

In the second phase, products of the first phase are conjugated with endogenous compounds, e.g., GSH, glucuronic, and/or sulfate acids, creating GSH conjugates, glucuronides, and sulfate esters that are, due to their hydrophilic nature, more efficiently eliminated from the body [84] (Figure 2).

In the third phase, an important role is played by ABC proteins, such as ATP binding cassette (e.g., multidrug resistance protein family—MRP) [85] (Figure 2).

### 4.1. Polymorphism of Genes Involved in B[a]P Metabolism and DNA Repair, Influencing Its Toxicity

Genetic polymorphism is associated with the presence of at least two different alleles at a given locus with a frequency greater than 1% in a population. We distinguish, among others: single nucleotide polymorphism and a variable number of tandem repeats polymorphism. The polymorphism of genes involved in the biosynthesis of enzymes implicated in the metabolism of xenobiotics concerns: cytochrome P450, flavin-containing mono-oxygenase, peroxidase, and carboxylesterase, as well as GST, UDP-glucuronosyltransferase, sulfotransferase, N-acetyltransferase, and methyl transferase, as well as quinone oxidoreductase and epoxide hydrolase of nontransferases of detoxification enzymes [86].

The main cytochromes P450 implicated in the formation of diols and diol-epoxides are CYP1A1, CYP1A2, and CYP1B1. The cytochrome P450 family has been well-studied in genetic polymorphisms’ and enzymes’ activity. Moreover, in recent years, its association with the development of various disorders has been widely discussed. The best-studied cytochromes are CYP2D6, CYP2C9, CYP2C19, and CYP1A1 [87]. Human cytochrome P450 1A1 is one of the most essential enzymes involved in human carcinogenesis, as it metabolizes several procarcinogens to active carcinogenic metabolites.

Certain polymorphisms in human CYPs and phase II enzymes (GST, uridine-5′-diphosphate, glucuronosyltransferases, and sulfotransferases) modulate cancer susceptibility [88]. In another metabolic pathway, B[*a*]P-7,8-dihydrodiol is oxidized to B[*a*]P-7,8-quinone by the enzymes from the aldo–keto reductase family, among which gene polymorphisms affecting NAD(P)H susceptibility have been identified. For example, quinone oxidoreductase-1 catalyzes reduction of B[*a*]P quinones to hydroquinones. These compounds can be reoxidized and induce ROS formation [6].

The genetic variability of cytochrome P450 (CYP) 2C9 and the presence of CYP2C9*2 and CYP2C9*3 cause significant interindividual differences in response to drugs and various xenobiotics. The presence of the polymorphic allele CYP2C9*2 (*Arg144Cys*) or CYP2C9*3 (*Ile359Leu*) significantly reduces the activity of the enzyme and B[*a*]P metabolism.

Ahmed et al. [89] studied frequencies of these polymorphisms in various ethnic groups living in Pakistan. Healthy volunteers (*n* = 467) from six major ethnic groups in Pakistan were tested. Twenty percent of the Pakistani population was found to have a genotype containing at least one low-activity allele.

Polymorphisms of various metabolizing genes related to the toxicity of B[*a*]P have been studied in many diseases, including oral squamous cell carcinoma (OSCC) [90]. The researchers evaluated the dominance of *CYP2 C9* polymorphism in healthy individuals and patients who suffered from oral squamous cell carcinoma (OSCC) and analyzed this disease risk development. Moreover, these researchers assessed the interaction between wild-type *CYP2 C9* with B[*a*]P and polymorphic variants. This clinical trial covered 46 OSCC patients and 46 healthy individuals. In the OSCC group, CYP2 C9*2 and CYP2 C9*3 polymorphisms constituted, respectively, 17.4% and 15.2%, and 8.7% and 6.5% in the control group, respectively. The OSCC group revealed a rise (statistically significant) in the frequency of *CYP2 C9* polymorphic variants in comparison to control individuals. It was found that B[*a*]P bound specifically to the changed catalytic site of the enzyme CYP2 C9*3. This research showed that a functionally important *CYP2 C9* polymorphism was present in OSCC patients, with a small rise in the risk of the disease in individuals who acquired these poor metabolizing variants.

Polymorphism may also apply to genes encoding DNA repair enzymes, which is related to the speed and efficiency of DNA repair, which may protect or expose a person to the effects of specific xenobiotics [91]. One of the studied polymorphisms is the nucleotide excision repair (NER) polymorphism, which is an important defense mechanism for an organism against mutagens and carcinogens, such as B[*a*]P. Genetic polymorphisms in *ERCC2/XPD*, a crucial component in NER, are believed to be linked with cancer risk development. Xiao et al. [92] examined cDNA clones carrying different *ERCC2/XPD* genotypes (*Lys751Gln*) that were inserted into the *ERCC2/XPD*-deficient (UV5) cell line in a controlled biological system. The wild-type AA (Lys) genotype was shown to be linked with a superior DNA repair capacity in comparison to its CC polymorphic genotype (Gln). These findings indicate that *Lys751Gln ERCC2/XPD* polymorphism influences the capacity of DNA repair upon exposure to carcinogens, such as B[*a*]P, in this controlled in vitro system, and can act as a biomarker enhancing the prognostic value of cancer development.

### 4.2. B[a]P Carcinogenesis Mechanisms Associated with Its Metabolism

**Figure 4:**
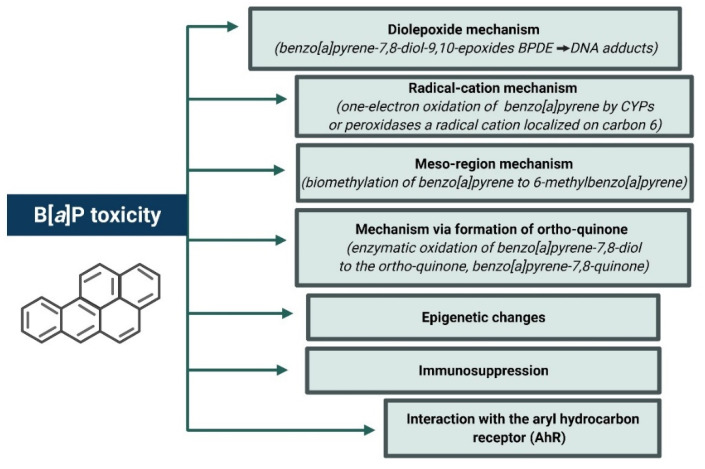
Mechanisms of B[*a*]P toxicity. Created with BioRender.com (accessed on 12 April 2022).

The explanation of the underlying mechanisms of B[*a*]P-induced carcinogenesis in animals is associated with pathways involved in the metabolism of B[*a*]P, two of which appear to be the most important pathways of dioxides and radical cations (Figure 4) [6].

The diol-epoxide mechanism for B[*a*]P is characterized by a sequence of metabolic transformations: B[*a*]P → B[*a*]P-7,8-oxide (by CYP1A1 and CYP1B1) → B[*a*]P-7,8-diol (by epoxide hydrolase) → B[*a*]P-7,8-diol-9,10-epoxides (by CYP1A1 and CYP1B1) [93]. Diol-epoxides react with DNA, mostly with purine bases, deoxyguanosine, and deoxyadenosine. Each diol-epoxide can create cis and trans adducts, which provides a total of 16 possible B[*a*]P-7,8-diol-9,10-epoxy DNA adducts, the most common of which is the *N*^2^-deoxyguanosine adduct (+)-*N*^2^-10S-(7R, 8S, 9R-trihydroxy-7,8,9,10-tetrahydroB[*a*]P)-yl)-2′-deoxyguanosine (BPDE-deoxyguanosine). The metabolite 7β,8α-dihydroxy-9α,10α-epoxy-7,8,9,10-tetrahydro benzo[*a*]pyrene (BPDE) participates in the production of this adduct. BPDE can create both unstable (so-called “depurinating”) and stable DNA adducts [94].

In vivo, anti-B[*a*]P-7,8-diol-9,10-oxide forms stable adducts, which are created mainly with guanines as observed in various species and organs [6]. It was found that in the lung tissue of mice exposed to B[*a*]P and anti-B[*a*]P-7,8-diol-9,10-epoxide-*N*^2^-deoxyguanosine, adducts were formed, and B[*a*]P-induced lung tumors showed G → T and G → A mutations in the Ki-Ras gene at the codon 12 [95]. In turn, skin tumors appeared in mice exposed to B[*a*]P had G → T mutations in codon 13 and A → T mutations in codon 61 of the Ha-Ras gene [94].

The radical-cationic mechanism consists of single-electron oxidation of B[*a*]P by CYP or peroxidase to a radical cation located at carbon 6. The presence of such metabolite and its adducts covalently linked with guanine (at carbon *C*^8^ and nitrogen *N*^7^) and adenine (at nitrogen *N*^7^) were shown in the skin of mice. These adducts are unstable and are believed to produce apurinic sites [94].

The mesoregion mechanism, on the other hand, is associated with biomethylation of the mesoregion and oxidation of benzyl. Biomethylation of B[*a*]P to 6-methylB[*a*]P takes place, and S-adenosylmethione is a carbon donor. Subsequent transformations of this metabolite result in the formation of 6-[(sulfoxide))methyl] B[*a*]P, which creates DNA adducts in vivo. The occurrence of these adducts was demonstrated in the rat liver [96].

Aldo–keto reductase enzymes play an important role in the mechanism through the formation of ortho-quinone/ROS. They oxidize B[*a*]P-7,8-diol to orthoquinone(B[*a*]P-7,8-quinone) [97] that may subsequently react with DNA, yielding both unstable and stable depurination adducts with DNA in vitro. It can also undergo repetitive redox cycles that generate ROS, provoking DNA oxidation. It was shown that in human A549 lung cancer cells, B[*a*]P-7,8-quinone increased 8-oxo-deoxyguanosine adducts formation, as well as induced the formation of DNA-strand breaks.

B[*a*]P and other PAHs can bind to the AhR nuclear complex, which may activate cytochrome P450, and in the consequence, lead to alterations in *CYP* gene transcription. It has been found that AhR knockout mice were resistant to B[*a*]P-induced oncogenesis [98].

#### 4.2.1. Oxidative Stress and Apoptosis as a Result of Increased Expression of Selected Genes and Increased CYP Activity

The activity of both CYP and peroxidases may contribute to the formation of radical cations as a result of single-electron oxidation. These cations constitute a separate group of terminal carcinogenic metabolites. The activity of CYP also produces a superoxide anion, which may contribute to oxidative stress [99].

Gao et al. [100] pointed to an important pathway in which B[*a*]P influenced ROS formation by affecting ROS/HIF-1α/HO-1 signaling (heme oxygenase 1—HO-1). They assessed the impact of B[*a*]P (1.5 µM and 25 µM) on A549 and MCF-7 cancer cells and noticed an increase in the ROS level and changes in HIF-1α and HO-1 in tested cancer cells.

Other studies have also shown induction of oxidative stress and apoptosis to be the mechanisms of B[*a*]P action. Elfawy et al. [101] observed that B[*a*]P induced morphological and physiological abnormalities in the zebrafish skeleton (head, spine, and tail) due to induced oxidative stress leading to apoptosis.

**Figure 5:**
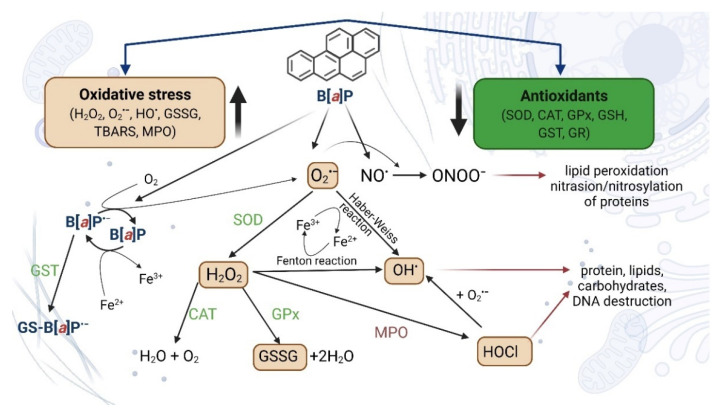
Environmental pollutants, such as B[*a*]P, affect the balance between the production of reactive intermediates and the biological system of ROS detoxification. The presence of B[*a*]P in the cell environment induces various molecular changes, such as lipid peroxidation or protein oxidation. B[*a*]P^·−^, anion B[*a*]P radical; O^·−^_2_, superoxide anion; H_2_O_2_, hydrogen peroxide; OH^•^, hydroxyl radical; HOCl, hypochlorous acid; NO, nitric oxide; ONOO, peroxynitrite; GS-B[*a*]P, glutathione-B[*a*]P conjugate; GSSG, glutathione disulfide (oxidized glutathione dimers); GR, glutathione reductase; GST, glutathione-S-transferase; CAT, catalase; SOD, superoxide dismutase; GSH, glutathione (reduced state); MPO, myeloperoxidase; TBARS, thiobarbituric acid reactive substances [107,108,109,110]. Created with BioRender.com (accessed on 12 April 2022).

Many publications have shown that B[*a*]P causes alterations in redox status and contributes to oxidative stress. B[*a*]P has been shown to decrease the activities of antioxidant enzymes, such as catalase (CAT), glutathione peroxidase (GSH-Px), superoxidative dismutase (SOD), GST, and glutathione reductase (GR), probably by changes in their expression. B[*a*]P also decreases the level of reduced glutathione (GSH), vitamin C, and vitamin E, as well as increases the level of lipid peroxidation and protein carbonyl formation, as well as increases the expression of *CYP1A1* and proinflammatory cytokines. Metabolites of B[*a*]P (mainly BPDE) and ROS created during transformation of B[*a*]P are the most important contributors to the oxidative effects provoked by this substance [102,103,104,105,106] (Figure 5).

**Figure 6:**
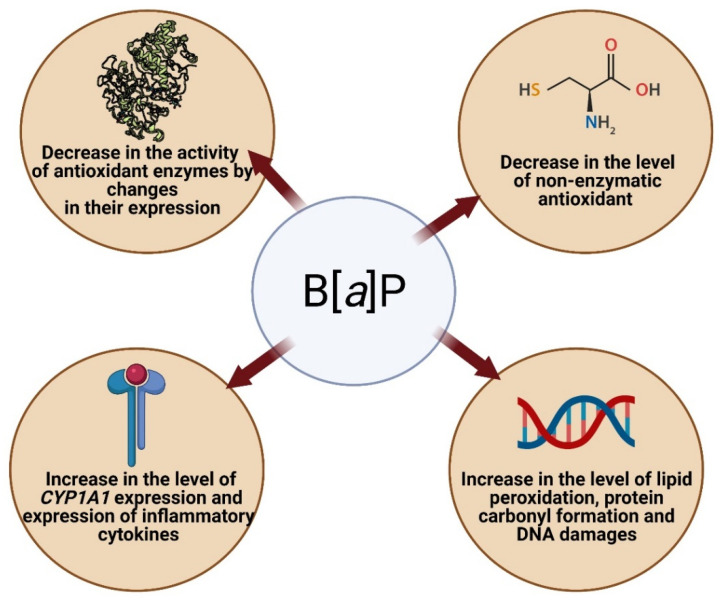
Pro-oxidative mechanisms of B[*a*]P action. Created with BioRender.com (accessed on 12 April 2022).

Bukowska and Duchnowicz [103] described in their review that B[*a*]P can increase AhR translocation and, as a consequence, increases levels of CYP450 and other proteins involved in B[*a*]P metabolism. As the result, increased BPDA-DNA adduct levels and other DNA damage can occur. Moreover, B[*a*]P has been reported to increase gene expression associated with inflammation and oxidative stress, as well as to deplete the expression and the activities of antioxidative enzymes, i.e., CAT, GPx, SOD, GST, and GR. Furthermore, B[*a*]P depleted nonenzymatic antioxidants levels, including GSH, Vit C, and Vit E. Metabolites of B[*a*]P (mainly BPDE) have been shown to induce ROS formation, as well as cause lipid peroxidation and protein carbonylation (Figure 6) [102,103,104,105].

In another study, Lin et al. [105] noticed that maternal exposure to PAHs and B[*a*]P was a risk factor for neural tube defects (NTD). The scientists suggested that oxidative stress and apoptosis could have been implicated in the mechanism of these changes. These researchers showed that intraperitoneal injection of B[*a*]P to the embryo after day 7 at the dose of 250 mg/kg induced NTD (incidence 13.3%) in ICR mice. The exposure to B[*a*]P significantly increased the expression of oxidative stress genes, i.e., *CYP1A1*, *SOD1*, and *SOD2*, with simultaneous suppression of *GPX1*. Enhanced apoptosis and a higher expression of caspase-3 were determined in the neural epithelium of embryos of B[*a*]P-treated mice.

Another mechanism presenting the oxidative effects of B[*a*]P was shown by Allmann et al. [111]. Transcriptional regulation of DNA repair is of paramount importance in restoring DNA integrity following genotoxic stress. These researchers showed that B[*a*]P activated a response to cellular DNA damage, causing transcriptional repression of the mismatch repair (MMR) genes (*MSH2*, *MSH6*, and *EXO1*) and *RAD51*, the central component of homologous recombination repair (HR), causing downregulation of MMR and HR. DNA repair repression was also reported after treatment with the metabolite of B[*a*]P—BPDE—and after ionizing ir-radiation, and also occurred as a result of ir-reversible cell cycle arrest induced by p53/p21, signifying the onset of cell aging. The suppression of MMR and HR is an early event during genotoxic stress-induced aging. It was proposed that sustained downregulation of DNA repair may participate in maintaining the aging phenotype that is associated with the accumulation of ir-reparable oxidative DNA damage [111].

#### 4.2.2. Oxidative Stress and Neurotoxicity

Some studies have documented a neurotoxic potential of B[*a*]P for the nervous system. Strong lipophilicity of B[*a*]P and its ability to bioaccumulate in lipid-rich tissues determine that this toxicant is particularly dangerous for the brain [112,113]. B[*a*]P has been found to cross the blood–brain barrier [114] and increase PAHs levels, as well as the levels of PAHs potentially reactive to biotransformation products in the brain [113].

The exposure to B[*a*]P also resulted in a progressive decline in spatial learning/memory and exploratory behavior in APP/PS1 and WT mice [115]. The exposure to B[*a*]P also enhanced neurodegeneration induced by the aging Aβ peptide in primary cultures of glial neurons acting through enhancement of the oxidative stress derived from NADPH oxidase. The research revealed that chronic exposure to B[*a*]P caused, enhanced, and exacerbated progression of Alzheimer’s disease (AD) in which the main pathogenic factor included increased inflammation of the nervous system and oxidative damage from NADPH oxidase.

Grova et al. [116,117] demonstrated that repeated intraperitoneal exposure of adult mice to B[*a*]P for 10 days caused behavioral abnormalities, such as anxiety and short-term memory and motor activity losses in these animals. These changes correlated with the level of oxidative stress and changes in the expression of N-methyl-D-aspartate (NMDA) Nr1 and Nr2a subunit mRNA in the hippocampus and in the frontal and temporal cortexes.

In another study, [118] B[*a*]P (20 and 200 mg/kg/day) was repeatedly given to mice, causing a significant overexpression of cytochrome. An elevated expression of *Cyp1A1*/*Cyp1B1* was noted in two of the three brain regions, showing the brain’s capability to metabolize B[*a*]P alone. Moreover, mice anxiety reduction was noted in the elevated plus maze and the hole board apparatus. This work also showed that B[*a*]P induced dose-dependent changes in NMDA subunits’ (Nr1 and Nr2a/Nr2b) expression in regions implicated in cognitive processes. The researchers also determined B[*a*]P metabolites, such as 9-OH-B[*a*]P and 7,8-diol-B[*a*]P in serum in the concentrations at which cognitive damage in mice was noticed.

Guo et al. [108] studied the effect of B[*a*]P at 10 and 100 μg/L on clam *Tegillarca granosa* in laboratory conditions and observed a neurotoxic potential of this substance, which was associated with inhibition of acetylcholinesterase and choline acetyltransferase activity. Moreover, significant DNA hypomethylation was observed, which was positively correlated with changes in the activity of tested enzymes.

#### 4.2.3. The Role of AhR Receptor in Toxicity and Carcinogenicity of B[*a*]P

AhR is a ligand-activated transcription factor that responds to various chemicals, such as chemical carcinogens. AhR is capable of inducing numerous genes implicated in immune regulation, detoxification, and proliferation. In respect to its capacity to recognize numerous chemical substances in the environment, it is often referred to as an environmental sensor.

**Figure 7:**
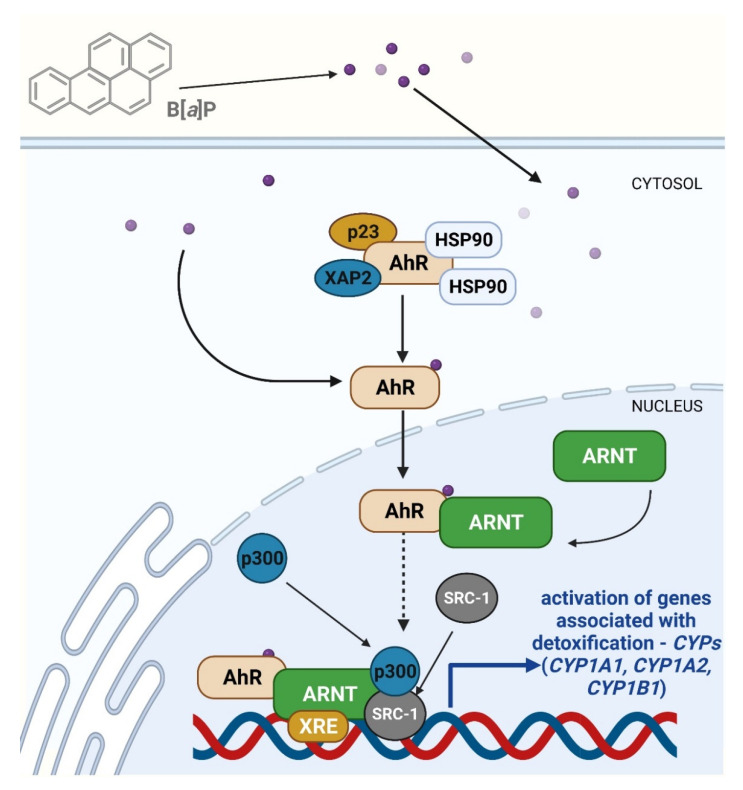
The aryl hydrocarbon receptor (AhR) is a ligand-activated transcription factor located in the cytosol. Inactive AhR occurs as a complex bonded with protein HSP90 (heat shock protein 90), p23 co-chaperone, and protein XAP2 (aryl hydrocarbon receptor-interacting protein, known also as AIP) or arachidonic acid (ARA). Binding AhR with ligand (B[*a*]P) leads to translocation of the active form of receptor to the nucleus and binds it with AhR receptor nuclear translocator (ARNT). Attachment of specific transcription factors (xenobiotic response element, XRE; histone acetyltransferase p300, steroid receptor coactivator-1, SRC-1) triggers gene machinery leading to activation of detoxification pathways (mainly by cytochrome P450 activation, CYPs). Intensification of ROS production can be correlated with DNA damage, mutation (mainly via PBDE), and carcinogenesis [122]. Created with BioRender.com (accessed on 12 April 2022).

B[*a*]P activates AhR and induces its translocation from the cytoplasm to the nucleus. The ligand-free AhR forms a cytosolic protein complex with the molecular chaperone HSP90, cochaperone p23, and XAP2 in the cytoplasm [119]. When exogenous ligands bind and cause AhR activation, it dissociates from the complex and migrates to the nucleus, where it dimerizes to create a complex capable of binding to the aryl hydrocarbon receptor nuclear translocator (ARNT). This complex activates the expression of a group of genes, such as the Ah gene battery, including cytochrome *P450 1A1* (*CYP1A1*), *CYP1A2*, and *CYP1B1* [120]. Importantly, the axis B[*a*]P-AhR-CYP1A1 additionally generates ROS and activates proinflammatory cytokines [120] (Figure 7).

B[*a*]P has been shown to increase mRNA expression of both *AhR* and *CYP1A1* genes in vivo, which suggests that it can play a role in carcinogenesis. In the study of Chang et al. [121], rats were injected intraperitoneally with B[*a*]P at 5, 10, and 15 µg/kg, and the mRNA expression of both the *AhR* and *CYP1A1* genes was determined after 24, 48, and 72 h. It was revealed that the mRNA expression of *AhR* and *CYP1A1* genes increased with B[*a*]P exposure in a dose-dependent manner.

AhR is contained in all tissues, particularly in the liver, adipose tissue, and bronchial epithelial cells. Therefore, AhR plays a role in chronic bronchitis, asthma, and chronic obstructive pulmonary disease (COPD), and has also been shown to play an important role in the development of lung cancer [123]. AhR plays a crucial role in the metabolism of B[*a*]P by affecting the expression of *CYP 450* and the regulation of numerous genes’ expression that contributes to the initiation, promotion, and progression of lung cancer. AhR participates through physical association with the nuclear factor kappa-light-chain-enhancer subunits of activated B cells (NF-κB), as well as influences downstream signaling pathways, which are involved in controlling lung cancer initiation and promotion. Recent data have suggested that interactions with additional transcription factors, such as nuclear factor-erythroid 2 (NF-E2), p45-related factor-2 (Nrf2), and estrogen receptor (ER), exist [123].

AhR activation has a variety of downstream effects that determine carcinogenesis, inflammation, DNA adduct formation, cell proliferation, and loss of cell-cell adhesion. In addition to its role in detoxification, a recent study has also shown new roles for AhR in cancer biology. Indeed, differential expressions of *AhR* have been noticed in various tumors in comparison to normal tissue. Such altered states of *AhR* expression participates in the pro- or antitumor action of cells depending on their condition [124]. A genomewide association research work of skin squamous cell carcinoma (SCC) also found AhR as a new site of susceptibility [125]. As reported by Hidaka et al. [120], AhR is a susceptibility factor for squamous cell carcinoma and a prognostic factor for melanoma and Merkel cell carcinoma. Additionally, it was noticed that carcinogenic effects caused by ultraviolet (UV) ir-radiation and carcinogenic substances are partly modulated by AhR, which regulates inflammation and apoptosis, DNA repair induced by UV, as well as activation of metabolism of chemical carcinogenic compounds. Additionally, AhR has been shown to modulate the effectiveness of therapeutic agents for melanoma. Activation of AhR triggers the expression of gene resistance to inhibitors of the mutant proto-oncogene V600 B-Raf and serine-threonine kinase in melanoma, and an increase in the expression of apoptosis protein 1 (PD-1) in T cells infiltrating tumors that surround the melanoma.

Another example of the carcinogenic effects of B[*a*]P involving metabolic reprogramming is the study conducted by [126]. These authors investigated the mechanism of AhR metabolic reprogramming associated with malignant transformation in BEAS-2B lung cells exposed to B[*a*]P. They observed that 52 metabolites were substantially changed in BEAS-2B cells exposed to B[*a*]P. It was also revealed that numerous metabolic pathways were substantially altered in B[*a*]P-exposed cells. It must be noted that most amino acids levels were substantially decreased, whereas the levels of most fatty acids were substantially increased in BEAS-2B cells exposed to B[*a*]P. Moreover, amino and fatty acids levels were associated with the levels of the aforementioned metabolites and AhR signaling after treatment with B[*a*]P. These researchers also found a reduction in peroxisome proliferator-activated receptor signaling and a rise in fatty acids imported by the FATP1 transporter in BEAS-2B cells treated with B[*a*]P.

Recently, Lou et al. [127] studied whether B[*a*]P regulates the metabolism of lipids via AhR. In this study, C57BL/6 mice were treated with B[*a*]P for 12 weeks to determine changes in serum lipids as well as glucose and insulin resistance. They observed that B[*a*]P inhibited weight gain, depleted lipid content, elevated lipid levels, and depleted glucose and insulin tolerance in tested animals. B[*a*]P also decreased *C/EBPα*, *PPARγ*, *FABP4*, *PGC-1α*, and *PPARα* expressions and increased *NF-κB*, *MCP-1*, and *TNF-α* expressions by activation of AhR. The researchers concluded that B[*a*]P was capable of inhibiting the synthesis and oxidation of fats, whereas triggering inflammation by activating AhR led to white adipose tissue dysfunction and metabolic complications.

## 5. Adverse Effects Observed in In Vitro and In Vivo Studies

### 5.1. Genotoxicity and Carcinogenicity

Cancer cells act differently than normal cells in many cellular processes. One of the main features of cancer is the Warburg effect, which is the glycolytic reprogramming that allows cancer cells to survive and proliferate. Hardonnière et al. [128] studied the metabolism of mitochondria and showed that B[*a*]P promoted the metabolic reprogramming characteristic of neoplastic cells. Cellular respiration was reduced, and lactate production was elevated. Observed alterations were associated with changes in the cycle of the tricarboxylic acid that include dysregulation of the mitochondrial complex II. The glycolytic shift depended on Na^+^/H^+^ exchanger activation and was discovered to be a crucial factor in the survival of the cells exposed to B[*a*]P.

Słowikowski et al. [129] indicated a possible carcinogenic synergy between estrogens, especially E2 and B[*a*]P. These researchers suggested that the combined effect of disturbed estrogen production in cancer cells due to B[*a*]P action may explain the increased aggressiveness and rate of development of lung cancer.

On the other hand, Tseng et al. [130], in the study with an AhR antagonist, found that B[*a*]P was also acting independently of the AhR pathway and suggested an alternative pathway of B[*a*]P toxicity. They showed that B[*a*]P induced fibrotic alterations with the depletion of α-1,6-fucosylation in lung stem cells (CD54 + CD157 + CD45), as well as observed that B[*a*]P disturbed spheroid formation and podoplanin expression in investigated lung stem cells, indicating that it inhibited differentiation in these cells. The observed changes indicated an increased risk of lung dysfunction and cancer development.

Genotoxicity of B[*a*]P in Rainbow Trout was assessed by Fanali et al. [131]. Young Rainbow Trouts were tested after exposure to B[*a*]P at 2 mg/kg. Among others, ethoxyresorufin-O-deethylase activity, as a biomarker of exposure to chemical substances, was assessed and a genotoxicity assay was performed. The obtained results indicated an increase in ethoxyresorufin-O-deethylase activity and an increased frequency of micronuclei formation, which may show a genotoxic potential of B[*a*]P in this fish species.

### 5.2. Epigenetic Effect

DNA methylation is a crucial gene regulation process that is sensitive to the action of environmental pollutants. A variety of xenobiotics have been identified as epigenetic modifiers, which are capable of inducing epimutations, particularly by influencing DNA methylation patterns [132]. In vitro studies have shown that B[*a*]P, a substance of carcinogenic potential and toxic for development and reproduction, is also an epigenetic modifier.

**Figure 8:**
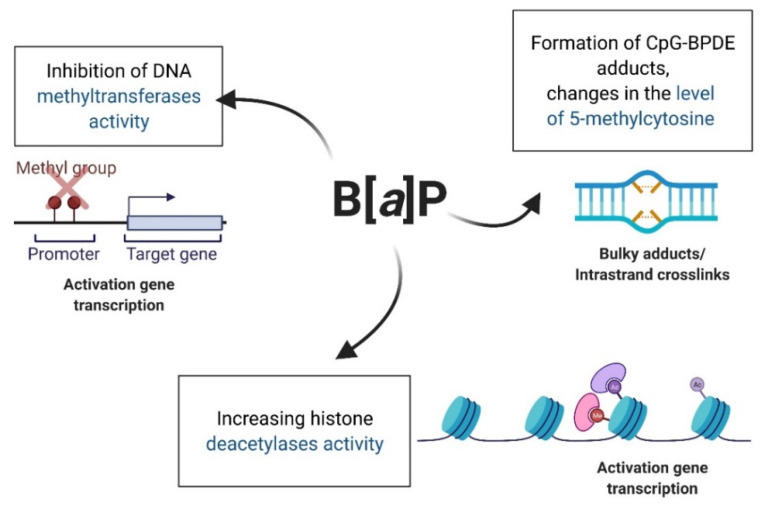
Mechanism of epigenetic changes induced by B[*a*]P. Created with BioRender.com (accessed on 12 April 2022).

B[*a*]P exhibits epigenotoxicity because it disrupts the processes of methylation of the entire epigenome [133,134,135], as well as promoters of individual genes [136,137]. B[*a*]P also influences the expression of histones [137,138] and induces the expression of miRNAs [139]. Bukowska and Sicińska [5] presented in their review that the mechanism of epigenetic changes induced by B[*a*]P was mainly associated with the creation of CpG-BPDE adducts, between B[*a*]P metabolite—BPDE—and CpG that led to alterations in the level of 5-methylcytosine [133,134,135]. Moreover, B[*a*]P has been shown to be able to inhibit the activities of DNA methyltransferases [140,141] and increase the activities of histone deacetylases (HDACs) [137] (Figure 8).

Recent studies have revealed that ancestral exposure to B[*a*]P causes intergenerational osteotoxicity in nonexposed F3 offspring. The consequences of environmental pollution with B[*a*]P/PAHs can thus be serious and may need reassessment. Mo et al. [142], in a review, suggested that transgenerational inheritance of osteotoxicity in fish, induced by ancestral exposure to B[*a*]P, is mediated by epigenetically deregulated bone miRNA/genes. Harmful effects of B[*a*]P on aquatic animals and their offspring have been confirmed by subsequent studies. Yin et al. [143] examined the offspring of sea medaka (*Oryzias melastigma*) treated with B[*a*]P at 0.5 mg/L and observed disturbed circadian oscillations and severe damage to DNA. Many clock-related genes, including per1 and p53, were substantially modulated in the offspring. Moreover, after paternal exposure to B[*a*]P, offspring had more severe damage to DNA and a higher hypermethylation degree than after maternal exposure. F1 larvae from parents exposed to B[*a*]P were more sensitive to B[*a*]P exposure, showing that expression of immunological and metabolic genes was substantially increased.

### 5.3. Epigenetic and Carcinogenic Effect of Benzo[a]pyrene in Epidemiological Studies—Exposure to B[a]P Present in PAH Mixtures

The International Agency for Research on Cancer (IARC) has classified B[*a*]P as “carcinogenic to humans” (Group 1) [6]. The carcinogenic effect of PAHs, including B[*a*]P, has been confirmed by numerous scientific studies. For a long time, various studies have indicated an increased risk of development of lung, skin, and bladder cancer in humans exposed to PAHs [144,145,146,147]. Recent research works have also implied that the exposure to PAHs is associated with increased risk of larynx, kidney, prostate, breast, blood (leukemia), brain, and colon cancers [11,13,148,149].

B[*a*]P, and particularly its metabolites, form DNA adducts, which lead to mutations. TP53 gene mutations have been shown to be common in the lung cancer of smokers, while they less frequently appeared in nonsmokers. These mutations are G → T transversions with hotspots at codons 157, 248, and 273 [150]. The active metabolite-anti-B[*a*]P-7,8-diol-9,10-oxide produces a spectrum of unique TP53 mutations, which differ from those observed in cancers, which are not linked with smoking. Similar G → T mutations were observed in lung tumors in nonsmokers whose tumors were linked with PAHs exposure from smoke produced by the combustion of coal in unventilated homes. Mutations were clustered in codons rich in CpG 153–158 of the TP53 gene and codons 249 and 273 [151].

Widziewicz et al. [152] examined the geographic distribution of lung cancer incidence in Poland and found that emission of B[*a*]P associated with PM10 from domestic heating contributed to the risk of lung cancer mainly in areas outside cities and agglomerations. When B[*a*]P concentrations were averaged across provinces, the exposure to B[*a*]P turned out to account for up to 31% of all cases of lung cancer.

Amadou et al. [13] assessed the relationship between exposure to airborne B[*a*]P and breast cancer risk. In the case–control study, 5222 women with breast cancer and 5222 appropriate controls were examined. The annual exposure to B[*a*]P of subjects was evaluated using the chemical-transport model and was assigned to geocoded home addresses of participants for each year from 1990 to 2011. This research elevated the known breast cancer risk connected with cumulative exposure to B[*a*]P, which varied with the status of menopause, the status of hormone receptors, and the degree of differentiation in breast cancer.

Epidemiological studies in human cohorts have implied correlations between PAHs exposure and a disturbed status of methylation in the offspring [133,134,135]. It has been suggested that global hypomethylation, as well as hypermethylation of specific genes, are connected with the development of cancer and other human disorders. Experimental data have shown that B[*a*]P adducts with DNA may regulate methylated genomic regions. Early embryonic development can be a period of particular susceptibility to B[*a*]P exposure (present, e.g., in cigarette smoke, as shown above), which results in an increased number of DNA adducts, as well as in disturbed DNA methylation.

Suter et al. [135] implied that frequent perinatal exposure (such as maternal smoking) dysregulates placental methylation in a CpG site-specific manner that correlates with substantial alterations in gene expression along signature pathways. It has been observed that the expression of 623 genes and the methylation of 1024 CpG dinucleotides were changed in smokers, with only 38 CpG revealing differential methylation (difference in methylation of ≥10%).

Herbstman et al. [133] also conducted an epidemiological study which assessed the effect of prenatal PAHs, including B[*a*]P, exposure on genomic DNA methylation in umbilical cord blood and assessed the associations between levels of methylation and the occurrence of PAHs–DNA adducts. They found that prenatal PAHs exposure was connected with lower global DNA methylation in umbilical cord blood cells and that the levels of global methylation were positively correlated with the occurrence of adducts in cord blood. The researchers suggested that PAHs exposure was sufficient to alter the global methylation level in the tested population. A similar aspect of this research was undertaken by Joubert et al. [134]. These workers determined the entire epigenome methylation in newborns’ umbilical cord blood in the context of maternal smoking during pregnancy. They found a number of genes with methylation alterations appearing at birth in babies whose mothers smoked during pregnancy. They proved that DNA methylation was statistically significant for the whole epigenome for 26 CpG mapped to 10 genes.

### 5.4. Effects on Reproduction

#### 5.4.1. B[*a*]P Effect on Males

Spermatogenesis is a controlled process during which a mature sperm is produced from sperm stem cells in three major stages, i.e., the mitotic stage, meiotic stage, and maturation stage. Germ cells undergo mutations during mitotic and meiotic divisions. Alterations in DNA sequence may be caused by cell treatment with xenobiotics during its entire life, but may also be inherited from mutations in spermatogonia stem cells, thus increasing the development risk of abnormalities or diseases in the offspring [153]. Damage to DNA related to B[*a*]P exposure was observed at all spermatogenesis stages, which were connected with a substantially reduced number of spermatozoids [154].

It was observed that subchronic exposure of adult male F-344 rats to inhaled B[*a*]P caused a depletion of testicular and epididymal functions in the studied animals [155]. Moreover, some studies have shown that the escalation of sperm hyperactivity occurs with an increase in the concentration of B[*a*]P due to premature capacitation [156].

Reddy et al. [157] assessed reproductive toxicity of B[*a*]P in adult male Wistar rats and concluded that chronic exposure to sublethal doses of B[*a*]P altered steroidogenesis and spermatogenesis, leading to depleted fertility in adult male rats. Rats received intraperitoneal injections containing B[*a*]P at doses of 1, 10, or 100 µg/kg b.w. each day for 60 days and were analyzed for fertility. At the end of dosing, the rats were euthanized and analyzed. It was found that the relative weights of the testes, epididymis caput, epididymal tail, seminal vesicle, and the prostate gland were substantially depleted in B[*a*]P-exposed animals. They have noted that daily sperm production and epididymal sperm count, mobility, and viability were substantially reduced in a dose-dependent manner in B[*a*]P-exposed rats. Moreover, a dose-dependent reduction in the activities of testicular steroidogenic enzymes was noted, whereas levels of serum testosterone were decreased in B[*a*]P-exposed animals. In silico research showed a binding affinity of B[*a*]P with the simulated StAR protein in the hydrophobic region of the tunnel and showed that the interaction of B[*a*]P with StAR could have led to reduced cholesterol transport to the mitochondria, which changes androgen synthesis in B[*a*]P-exposed rats.

A very interesting study was conducted by Jorge et al. [158], who assessed how peripubertal exposure to B[*a*]P in male rats could cause reproductive disorders in the offspring. B[*a*]P was given orally to male rats in the postnatal period 23–53 days at environmentally relevant doses (0, 0.1, 1, or 10 µg/kg/day). On day 90 of the postnatal period, exposed males were mated with untreated B[*a*]P females to conceive the next generation (F1). Paternal exposure to B[*a*]P depleted the body weight of the offspring and also resulted in a depletion in the relative anorectal distance in males. The exposure to B[*a*]P also accelerated the onset of puberty, as shown by an earlier vaginal opening and first estrus in the lowest dose group of females, as well as a delay in testicular prolapse and foreskin-separation age in males. In males, a reduction in the production of daily sperm, as well as disturbed sperm morphology, were observed. Moreover, testicular histology was changed, as shown by a depletion in the number of Leydig cells and the diameter of seminal vesicles. Females showed changes in estrus cycles and some fertility parameters, as well as histological changes in the ovaries and uterus. B[*a*]P changed the reproductive parameters of the F1 generation, which suggested that the peripubertal exposure of the F0 generation to this compound caused permanent modifications in the reproduction of these animals [158].

#### 5.4.2. Effect on Females

The ovarian function may be compromised by exposure to toxicants. Xenobiotics can affect the ovary itself, leading to impaired oocyte maturation and/or its destruction. The mechanism of B[*a*]P-mediated ovotoxicity may be indirect because oocytes are surrounded by follicular cells at all stages of development [159]. The loss of integrity of the follicle wall as a result of B[*a*]P exposure may adversely affect maintaining oocyte viability [160]. Extensive damage to the follicles can also impair the production of steroid hormones, which in turn affects the hormonal balance and leads to ovarian failure [161]. Lim et al. [162] noticed that developing ovaries are more vulnerable when compared with testes to prenatal exposure to B[*a*]P, which may be associated with the formation of higher B[*a*]P metabolites levels in female embryos.

In ovarian tissue, adducts of the B[*a*]P metabolite—BPDE—with DNA have been determined in oocytes and luteal cells in the ovaries of adult women exposed to cigarette smoke from smoking or passive smoking [163]. B[*a*]P has been proven to cross the placenta in mice, rats, and guinea pigs as a result of maternal injection, inhalation exposure, or dermal contact [164]. Treatment of pregnant mice or rats with B[*a*]P (inhalation) caused a reduction in the number of live pups at birth [165]. B[*a*]P injection into pregnant rats caused reduced fetal weight, as well as increased fetal mortality [166], whereas giving B[*a*]P by ingestion to pregnant females increased the number of stillborn young [167].

Yang et al. [168] investigated the mechanisms underlying endocrine and reproductive disorders in bivalves exposed to B[*a*]P. The analysis covered three reproductive stages (the proliferative, growth, and mature stages) of female *Chlamys farreri* scallops, which were exposed to B[*a*]P at 0.38 and 3.8 µg/L. This research showed a toxic impact of B[*a*]P on the gonadosomatic index, endocrine-related gene expression, circulating hormone levels, and ovarian histology. A substantial depletion in sex hormones, including testosterone, progesterone, and 17β-estradiol levels, was noticed in B[*a*]P-exposed *C. farreri* during phases of growth and maturity. These effects were linked to decreased steroidogenic enzymes expression, including 3β-hydroxysteroid dehydrogenase, *CYP17*, and *17β-HSD*, which were regulated by the upstream signaling pathway of adenylate cyclase and protein kinase. It was also found that ovarian estrogen receptor and caveolin-1 (cav-1) transcripts levels were depleted in *C. farreri*. Moreover, vitellogenin, a precursor of egg yolk involved in ovarian development, was downregulated by B[*a*]P [168].

#### 5.4.3. Effect on Fetal Development

Yamaguchi et al. [169] studied embryonic teratogenicity and developmental toxicity of B[*a*]P in Japanese Medaka (*Oryzias latipes*) after exposure to B[*a*]P at 0.1 and 1 mg/L for 2 h. Embryos exposed to B[*a*]P were found to reveal typical teratogenic and developmental effects, including cardiovascular and developmental abnormalities and curvature of the spine. DNA microarray analysis showed several unique upregulated genes, such as those connected with cardiovascular disease, various cellular processes, and neuronal development. These findings have suggested a potential teratogenicity and developmental toxicity of B[*a*]P.

Epidemiological studies have shown that increased PAHs, including B[*a*]P, exposure could be connected with a variety of abnormalities, including lower serum progesterone levels, reduced fetal growth [170], and a higher risk of early miscarriage, which suggests a possible disturbing effect of these toxicants on the reproductive process [171]. Population analyses have also indicated that mice exposed to B[*a*]P showed depleted fetal weight, length, and head circumference; preterm delivery; birth defects; and reduced growth [172]. Ye et al. [171] recently investigated the effects of B[*a*]P and its metabolite, BPDE, on human trophoblasts and miscarriages in mice. They found a dose-dependent incidence of abnormal murine fetuses starting from a dose of B[*a*]P of 0.1 µg/kg; at a dose of 2 mg/kg, no fetus was developed. Apoptosis in human trophoblasts was also demonstrated, which was associated with BPDE concentration. Moreover, BPDE from a dose of 0.5 μM caused an increase in Bax/caspase-3, and a decrease in Bcl-2 levels BPDE was also shown to inhibit, in a concentration-dependent manner, chorion explants’ migration from women who had undergone an elective abortion procedure, which is in agreement with depleted chorion explants’ migration from women who experience a recurrent loss of pregnancy. Moreover, in a dose-dependent manner, it reduced cell immigration in Swan 71 trophoblasts.

## 6. Impact on Virus Development

Smoking increases the pathogenesis of HIV-1 and reduces the response to antiretroviral therapy. Ranjit et al. [173] showed a ~three- to fourfold rise in the replication of HIV-1 in U1 cells and human primary macrophages after chronic treatment with B[*a*]P. They also noticed a ~30-fold rise in the expression of *CYP1A1* at the mRNA level, a ~2.5-fold rise in its enzymatic activity, and an increase in ROS level and cytotoxicity in U1 cells. Inhibition of the *CYP1A1* gene with siRNA and treatment with selective CYP inhibitors and antioxidants substantially depleted HIV-1 replication. Similarly, suppression of the NF-κB pathway with specific NF-κB inhibitors also substantially decreased HIV-1 replication. The authors suggested that B[*a*]P enhanced HIV-1 replication in macrophages through the CYP-mediated oxidative stress pathway followed by the NF-κB pathway.

Zhang et al. [174] investigated the effects of 17 PAHs on high-risk Human Papillomavirus (HPV16). They showed that the HPV16 E7 oncogene was expressed in primary cells extracted from the kidneys of young rats, which were treated with PAHs. B[*a*]P, dibenzo[*a,h*]anthracene, and indeno[1,2,3-*cd*]pyrene revealed the strongest potential for cotransformation in the renal cell system of young rats. Short-term exposure to B[*a*]P did not change the proliferation of C33A or CaSki cells; nevertheless, long-term exposure caused a dramatic rise in CaSki cell growth rate by 120–140%. In addition, the exposure to B[*a*]P changed the mobility and invasiveness of C33A and SiHa cells, but not of MCF7 cells. These results indicated that exposure to B[*a*]P may be a key cofactor in HPV-related cancer development. B[*a*]P can operate at all three stages: initiation, promotion, and progression.

## 7. Other Effects

B[*a*]P has been reported to be implicated in the accelerated mucus secretion in the airways and high mucin 5AC (*MUC5AC*) expression. Sun et al. [175] proved that B[*a*]P increased the levels of mRNA and MUC5AC proteins in airway epithelial cells. In turn, Tajima et al. [176] indicated that B[*a*]P-induced AhR receptor activation was implicated in a proinflammatory response to a respiratory allergy in a mouse model of allergic airway inflammation, and this process could have been mediated by the increased expression of IL-33 and eosinophil infiltration. Moreover, Choi et al. [177] reported that B[*a*]P strongly influenced nonatopic asthma, while there was no clear association with atopic asthma.

## 8. Summary

B[*a*]P is commonly found in the air, surface water, soil, food, dust, and cigarette smoke. Due to high hydrophobicity, B[*a*]P is present in high concentrations in aquatic sediments and soil, while low concentrations of this substance are present in surface waters. Outdoor air is often contaminated with B[*a*]P at concentrations which consistently exceed acceptable standards. In particular, inhaling indoor air containing very high B[*a*]P concentrations poses a serious health risk for children in school and kindergarten, as well as workers employed in aluminum factories or coke-oven facilities.

This substance is very toxic, and it is a group I carcinogen. B[*a*]P is also genotoxic, mutagenic, epigenotoxic, teratogenic, and neurotoxic, and it impairs fertility. The mechanism of B[*a*]P-action involves the formation of depurinating DNA adducts, generation of ROS, activation of AhR, and various epigenetic changes.

Undue pollution of the atmosphere with B[*a*]P is now one of the essential environmental health threats for numerous countries. Taken into consideration the current state of B[*a*]P air pollution, the governments of those countries must implement new measures to reduce B[*a*]P emissions into the atmosphere.

On the other hand, B[*a*]P that has already been introduced in the environment (particularly the presence in heavily contaminated soils), must be eliminated as soon as possible by the usage of bacteria strains capable of efficiently degrading this substance.

## Figures and Tables

**Table 1:** B[*a*]P levels in outdoor air.

Location	Concentration [ng/m^3^]	Reference
The European Union	7% of EU citizens live in areas with a tolerable risk level of 0.12 ng/m^3^	[12]
France	1 ng to 2.49 ng/m^3^	[13]
Thailand	0.052 and 0.095 ng/m^3^ in PM 2.5 fraction	[14]
Iberian Peninsula	exceeded target value of 1 ng/m^3^	[15]
Italy—Genoa	2 ng/m^3^ (along heavy traffic streets)	[16]
	14 ng/m^3^ (300 m from a coke oven)	
Poland—Cracow	4–10 ng/m^3^,	[17]
Tarnow	4–6 ng/m^3^	
Nowy Sacz	10–11 ng/m^3^	
China—Linzhou	5.1–20.2 ng/m^3^	[18]
Saudi Arabia—Makkah	0.082 ± 0.032 ng/m^3^ (occupationally exposed workers)	[10]
	0.044 ± 0.006 ng/m^3^ (unexposed group)	

**Table 2:** B[*a*]P levels in indoor air.

Location	Concentration	Reference
North China kitchens, indoor air	14.3 ± 23.0 ng/m^3^ (gaseous phase)	[29]
	6.7 ± 17.4 ng/m^3^ (particulate phase)	
China Yucheng City kitchens, indoor air	25.8 ± 10.6 ng/m^3^ (oil-based cooking)	[30]
	7.3 ± 4.6 ng/m^3^ (water-based cooking)	
Tibet Jokhang Temple, indoor air	18.5 ± 4.3 ng/m^3^	[31]
China Shanxi Provence schools, indoor air	0.05 ng/m^3^ (nonheating season)	[32]
	10.3 ng/m^3^ (heating season)	
Saudi Arabia, Jeddah’s schools, indoor air	163.87 ± 68.53 ng/m^3^	[33]
Poland, Silesia kindergartens, indoor air	3.7 ± 0.8 ng/m^3^	[34]
India, Shimoga, iron foundry	7.20 ± 1.11 μg/m^3^ (melting section) 45.37 µg/m^3^ (molding section)	[35]
Sweden, Aluminum manufacturing factories	14 μg/m^3^	[36]
United Kingdom, Coke oven facilities	3.3 μg/m^3^	[37]

**Table 3:** B[*a*]P levels in soils of various countries of the world since 2000.

Location	Land Type	Concentration (µg/kg)	Reference
Bangkok	Urban–tropical	5.5	[47]
Brazil	Forest–tropical	0.3	
New Orleans	Urban	276	[48]
Dalian, China	Traffic	388	[49]
	Park	71	
	Suburban	27	
	Rural	9	
United Kingdom	Rural	46	[50]
Norway	Rural	5.3	
Spain	Industrial–chemical	100	[51]
	Industrial–petrochemical	18	
	Residential	56	
	Rural	22	
Poland	Agricultural	30	[52]
Poland, Bialystok	Urban	300–900	[44]
USA, Cleveland	Municipal plots	280–5500	[45]
Russia, St. Petersburg	Parkland	220	[53]
	Residential	430	
	Industrial	340	
Turkey, Antalya Aksu region	greenhouse crops	2.31	[46]
Taiyuan	Urban	94.03	[42]
	Agricultural	65.57	
	Montane	16.60	
Antarctic Peninsula	Antarctic station territory	1.5	[54]
United Kingdom, Cities of Crimea	Alushta	60	[55]
	Yalta	139	
	Sebastopol	260	

## Data Availability

Not applicable.
